# Valorization of Winemaking By-Products: White and Red Grape Seed Oils Improve Glucose Consumption and Uptake In Vitro

**DOI:** 10.3390/molecules30091933

**Published:** 2025-04-26

**Authors:** Daniela Ganci, Federica Bellistrì, Manuela Mauro, Roberto Chiarelli, Francesco Longo, Serena Indelicato, Sergio Indelicato, Vito Armando Laudicina, Vincenzo Arizza, Mirella Vazzana, Claudio Luparello

**Affiliations:** 1 Dipartimento di Scienze e Tecnologie Biologiche Chimiche e Farmaceutiche (STEBICEF), Università di Palermo, 90128 Palermo, Italy; daniela.ganci01@unipa.it (D.G.); federica.bellistri@community.unipa.it (F.B.); manuela.mauro01@unipa.it (M.M.); roberto.chiarelli@unipa.it (R.C.); francesco.longo03@unipa.it (F.L.); serena.indelicato@unipa.it (S.I.); vincenzo.arizza@unipa.it (V.A.); mirella.vazzana@unipa.it (M.V.); 2 National Biodiversity Future Center (NBFC), 90133 Palermo, Italy; 3 Azienda Ospedaliera Ospedali Riuniti Villa Sofia Cervello, Chromatography and Mass Spectrometry Section, Quality Control and Chemical Risk (CQRC), 90146 Palermo, Italy; nondelicato@gmail.com; 4 Dipartimento di Scienze Agrarie, Alimentari e Forestali, Università di Palermo, 90129 Palermo, Italy; vitoarmando.laudicina@unipa.it

**Keywords:** fatty acids, carotenoids, chlorophyll, polyphenols, GLUT, HNF1α, IRS1, AKT, AMPK, PKCζ

## Abstract

The rising demand for alternative solutions to diabetes mellitus has prompted significant interest in the exploration of plant-derived anti-diabetic compounds, especially within a circular economy framework that seeks sustainable and profitable reuse options. In this context, red (RSGO) and white (WGSO) grape seed oils, by-products of Sicilian vineyards, were prepared, analyzed for their fatty acid, polyphenol, carotenoid, and chlorophyll content, and evaluated for their glucose-lowering ability on HepG2 cells. Utilizing cytochemical techniques, flow cytometry, and protein blotting, we explored the effects of non-toxic oil dilutions on (i) glycogen storage, (ii) glucose consumption/uptake, (iii) GLUT-2, GLUT-4, and hepatocyte nuclear factor-1α (HNF1α) expression levels, and (iv) AMP-activated protein kinase (AMPK), insulin receptor substrate-1 (IRS-1), AKT, and PKCζ phosphorylation states, which are involved in insulin-mediated and -independent regulation of GLUT-4 membrane exposure. RGSO and WGSO, despite adopting slightly varying molecular strategies, were both proven to be effective stimulators of glucose absorption and glycogenesis. Specifically, RSGO promoted GLUT-2 and GLUT-4 up-regulation, whereas the WGSO-induced effect was associated with an increase in GLUT-4 levels alone. Moreover, the oils activated both pathways responsible for GLUT-4 translocation. Therefore, these wine-making residues have substantial potential as anti-diabetic solutions, holding promise for integration into the biomedical and food sectors.

## 1. Introduction

*Vitis vinifera* (L.), i.e., the grapevine, is a fruit crop of significant economic relevance belonging to the Vitaceae family, which comprises 16 genera and about 950 species that thrive in temperate climates of Europe and in tropical regions [1]. Wine crafting leads to the creation of considerable waste accounting for roughly 30% of the total grapes utilized. This includes materials such as vine trimming, grape stems, grape pomace (a blend of skins, seeds, and stem fragments), and lees. Disposing of these by-products poses a major environmental issue. In the context of a circular economy that strives for sustainable and profitable reuse solutions, the investigation into wine-making residues as readily available sources of biomass and organic matter has been the focus of numerous studies [2].

Grape seeds constitute the majority of the pomace residue, representing 38–52% of the total dry weight [3]. Their oil’s composition is determined by the grape variety, environmental factors, and the seeds’ ripeness, and it is generally characterized by a richness in hydrophilic components, like phenolic compounds, as well as lipophilic components such as vitamin E, unsaturated fatty acids, and phytosterols [4,5]. These bioactive compounds within the oil play a prominent role in its health advantages, exhibiting a variety of effects, mostly observed in in vitro models, which range from antioxidant and anti-inflammatory to anti-tumor, anti-aging, anti-cholesterol, anti-platelet, antimicrobial, and anti-diabetic [4,6,7,8,9].

Type II diabetes mellitus is a multifaceted metabolic disease that poses a significant threat to human health. With lifestyle changes and the growing prevalence of obesity, the total number of adults aged 20–79 years diagnosed with this condition is expected to rise from 463 million in 2019 to 700 million by 2045. Diabetes mellitus is characterized by a permanently elevated blood glucose level and a compromised glucose tolerance, which occurs due to the failure of pancreatic β cells to adequately produce insulin, the presence of circulating insulin antagonists, or insulin resistance which involves reduced glucose uptake by peripheral tissues and increased hepatic glucose production [10]. The primary organ responsible for glucose metabolism and storage is the liver, which is also the main target for the molecular changes that impact glucose uptake and utilization. The essential problem associated with glucose metabolic dysfunction that occurs in diabetes mellitus is the reduced expression and functionality of the GLUT transporters. In hepatocytes, GLUT2 serves as the main carrier allowing the bidirectional transport of glucose via low affinity-binding mechanisms in an insulin-independent way and following a concentration gradient. Blood glucose levels and adipogenesis factors control its expression, and hepatocyte nuclear factor 1α (HNF1α) is recognized as a key stimulatory transacting factor that promotes human *GLUT2* expression [11,12]. Differently, the GLUT4 transporter, which is underrepresented in liver cells, acts as a high-affinity carrier that mediates the influx of glucose when insulin binds to its receptor, thereby activating the insulin receptor substrate-1 (IRS1)/PI3K signaling pathway. This may lead to a divergence by activating through phosphorylation two distinct downstream kinase effectors, i.e., protein kinase B, also known as AKT, and/or protein kinase Cζ (PKCζ), that triggers the translocation of GLUT4-containing intracellular vesicles from the perinuclear area to the plasma membrane [13,14,15]. Of note, the phosphorylation of IRS-1 at serine and threonine residues inhibits the signalization cascade, thus preventing the insulin-mediated accumulation of the carrier on the cell’s surface. In addition, research demonstrated that the translocation of GLUT4 can be also stimulated by the activation of the AMP-activated protein kinase (AMPK) signaling pathway, independent from insulin, following its phosphorylation [16,17].

Treatment strategies for type II diabetes mellitus focus on maintaining blood glucose levels within the appropriate range. Although numerous pharmacological agents are available for this objective, their administration is often linked to undesirable side effects. Therefore, there is an urgent requirement for new, reliable, and affordable anti-diabetic drugs. In this context, the interest in developing anti-diabetic compounds from plants has become prominent due to their cost-effectiveness, reduced adverse effects, and easy accessibility [18]. Nevertheless, it is crucial to conduct thorough scientific verification to confirm their suitability for medicinal use. The exploration of grape seed oil’s potential benefits for diabetes management has not been extensively studied to date. Evidence from in vivo experiments revealed that the administration of grape seed oil to diabetic rats or mice consuming a high-fat diet resulted in a significant reduction in hyperglycemia [19,20]. Likewise, the consumption of the oil has been proven to significantly lower serum glucose levels in overweight and obese female subjects [21].

Thus, focusing on the discovery of alternative sources for functional components within a circular economy and biorefinery framework, this work aims to explore the valorization of red grape seed oil (RSGO) and white grape seed oil (WGSO), by-products of Sicilian vineyards, as promising candidates for anti-diabetic formulations. For this purpose, the phytochemical compositions of the RSGO and WSGO under study were assessed and, by applying a combination of cytochemical, flow cytometric, and protein blotting techniques, the comparative effect of each oil on glycogen accumulation and glucose consumption/uptake by HepG2 liver cells was evaluated. This study also explored the impact of the oils on the protein expression of GLUT2 and -4 transporters alongside selected components of the GLUT signaling pathways.

## 2. Results

### 2.1. Phytochemistry of RGSO and WGSO

#### 2.1.1. Fatty Acids, Carotenoids, and Chlorophyll

As shown in Table 1, the fatty acid composition and ratios in the two grape seed oils exhibited comparable characteristics. In terms of abundance, the fatty acids were identified in the following order: linoleic acid (C18:2 ω6,9 cis, cis) was the most prevalent, followed by oleic acid (C18:1 ω9 cis), palmitic acid (C16:0), and, finally, stearic acid (C18:0). The relative abundance of all remaining fatty acids was below 1%. The distinction between RGSO and WGSO was primarily attributed to the differing levels of oleic and linoleic acids, with RGSO being more abundant in linoleic acid and WGSO in oleic acid. In addition, trace quantities of other fatty acids, including C20:2 and C:22, appeared exclusively in RGSO.

Concerning carotenoids and chlorophyll, Table 2 indicates that RGSO exhibited a significantly higher amount of carotenoids and was the only oil that contained a quantifiable amount of chlorophyll.

#### 2.1.2. Phenolic Compounds

A summary of the phenolic compounds identified via UHPLC-MS/MS analysis can be found in Table 3. Distinctive profiles, both qualitative and quantitative, of phenolic compounds were observed in the two grape seed oils. In particular, RGSO exhibited the highest levels of syringic acid and trans-hydroxycinnamic acid among the compounds analyzed. Additionally, hydroxytyrosol and olacein were identified, although their concentrations were below the limits of quantification. In WGSO, syringic acid and kaempferol were the most abundant polyphenols.

### 2.2. In Vitro Glucose-Lowering Activity of RGSO and WGSO

**Figure 1:**
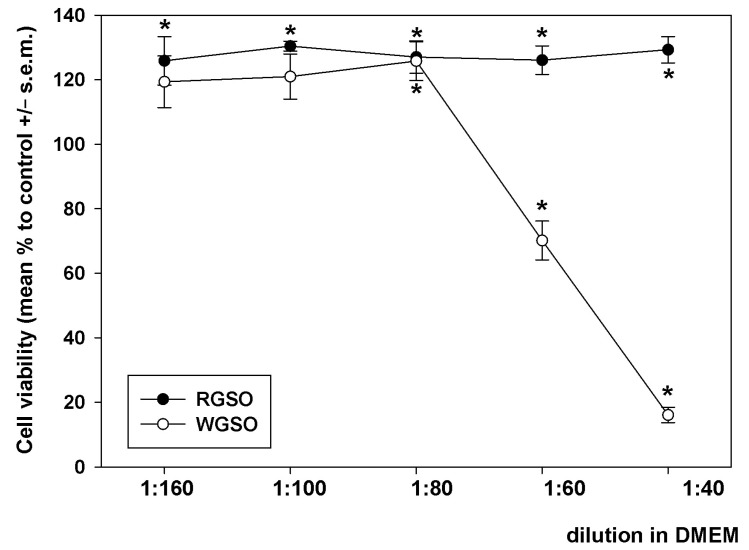
Dilution-response effect of RGSO and WGSO on the viability of HepG2 cells after 24 h of exposure. The error bars correspond to the standard error of the mean (s.e.m.) of three independent measurements. * *p* < 0.05 compared to control. Normality test passed.

Preliminarily, HepG2 cells were exposed to decreasing dilutions (from 1:160 to 1:40) of RGSO and WGSO in a complete DMEM medium for 24 h, and the analysis of cell viability was conducted via the MTT assay. As depicted in Figure 1, RGSO did not exhibit any cytotoxicity within the tested concentration range, but instead, it induced a limited yet steady increase in cell viability. Starting from 1:80, a dilution-dependent decline in cell viability occurred when cells were treated with WGSO. The analysis of the obtained dose-response curve identified 1:80 as the minimum non-inhibitory dilution (MNID), i.e., the lowest dilution that does not interfere with cell survival, and this was selected for all following experiments. For an appropriate comparison of the oils, although RGSO was ineffective in impairing cell viability, the identical dilution of this preparation was applied in parallel biological assays.

**Figure 2:**
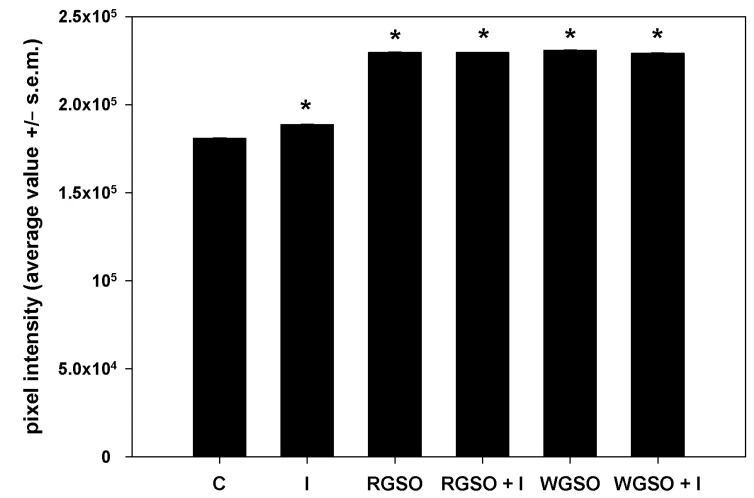
PAS staining intensity of HepG2 cell cultures treated with RGSO or WGSO MNIDs, with or without the addition of 10^−7^ M insulin, or the sole 10^−7^ M insulin (I) for 24 h. The error bars correspond to the standard error of the mean (s.e.m.) of three independent measurements. * *p* < 0.05 compared to the untreated control (C). Normality test passed.

To evaluate the effects of RGSO and WGSO MNIDs on glucose metabolism in HepG2 cells, we first examined the intracellular accumulation of glycogen in the presence of either oil, with or without the addition of 10^−7^ M insulin, or of the sole 10^−7^ M insulin. Following the PAS staining, images of different microscopic fields were taken, pixel intensities were calculated, and their average values were plotted in a bar graph (Figure 2). Prototypical micrographs of PAS-stained cell preparations under different experimental conditions are shown in Figure S1. The results demonstrate that, as expected, cells cultured for 24 h in the presence of insulin showed an increase in glycogen storage compared with controls. Interestingly, after exposure to both RGSO and WGSO, the amount of glycogen stained was higher than in cells treated with insulin alone. In addition, co-treatment with RSGO or WGSO plus insulin did not result in any significant additional up-regulation of intracellular glycogen storage.

**Figure 3:**
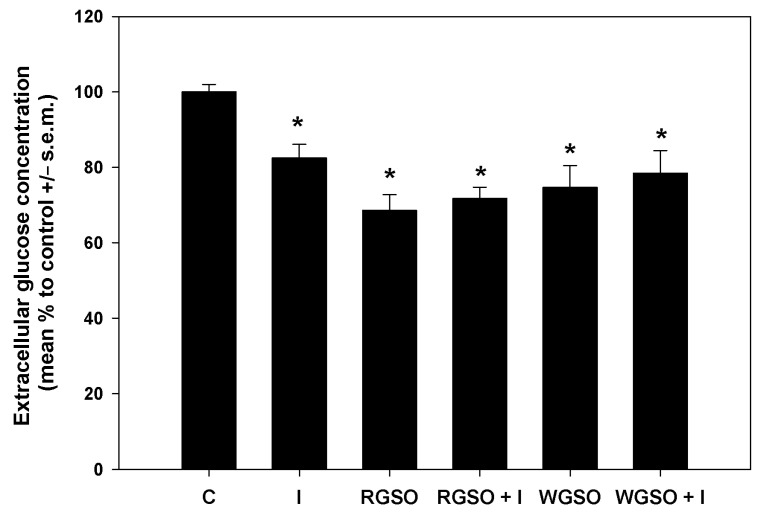
Extracellular glucose concentration in HepG2 cell cultures treated with RGSO or WGSO MNIDs, with or without the addition of 10^−7^ M insulin, or the sole 10^−7^ M insulin (I) for 24 h. The error bars correspond to the standard error of the mean (s.e.m.) of three independent measurements. * *p* < 0.05 compared to the untreated control (C). Normality test passed.

To delve deeper into the glucose-lowering properties of the oils, glucose consumption and glucose uptake were measured in the different experimental conditions. As shown in Figure 3, after a 24 h exposure to RGSO or WGSO MNIDs, the extracellular glucose concentration was reduced by 31.5 ± 4.2 and 25.3 ± 5.8% compared to the control, respectively, which is a greater decrease than the 17.6 ± 3.7% reduction achieved with 10^−7^ M insulin alone. Co-exposure to either oil and 10^−7^ M insulin did not yield a statistically significant synergistic effect, and the extent of glucose level reduction in the culture medium was similar to that achieved with each oil alone. The absence of statistical significance in the assays for extracellular glucose concentration and glycogen storage led to the exclusion of RGSO/insulin and WGSO/insulin co-incubation conditions from subsequent experimental investigations.

**Figure 4:**
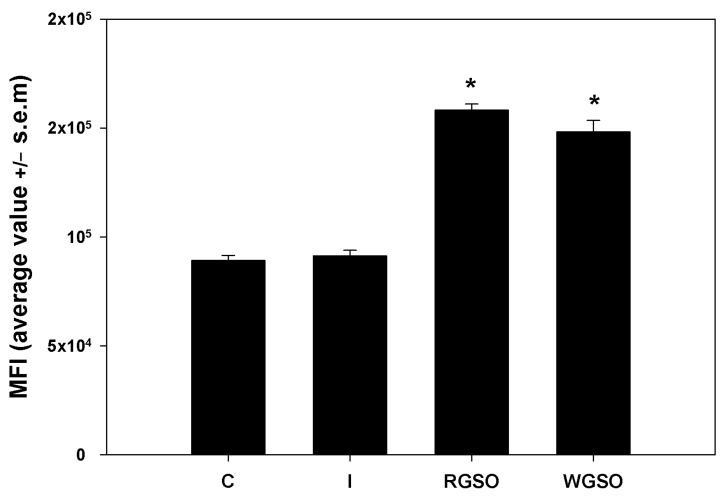
2-NBDG uptake by HepG2 cells treated with 10^−7^ M insulin (I), RGSO, or WGSO MNIDs for 24 h, evaluated as the MFI of the positive fraction. The error bars correspond to the standard error of the mean (s.e.m.) of three independent measurements. * *p* < 0.05 compared to the untreated control (C). Normality test passed.

To evaluate the short-term glucose uptake capabilities of HepG2 cells under the different culture conditions, cells were incubated with 2-NBDG, a non-metabolizable fluorescent glucose analog, for 1 h and immediately examined by flow cytometry. The mean fluorescence intensity (MFI) of the positive fraction was measured for each specific experimental condition. As depicted in Figure 4, the data reveal that exposure to both RGSO and WSGO produced a significant and rapid response leading to the up-regulation of 2-NBDG uptake by approximately 1.7- and 1.8-fold, respectively, when compared to the control. Conversely, as reported elsewhere (Abruscato et al., submitted), the exposure to 10^−7^ M insulin alone failed to produce a significant short-term increase.

### 2.3. Effect of RSGO and WSGO on GLUT-2 and -4 Transporters and Associated Regulatory Factors

To shed some light on the specific mechanisms by which RGSO and WSGO impact glucose metabolism, we examined the expression levels of the insulin-insensitive GLUT-2 and insulin-stimulated GLUT-4 transporters. The investigation also included the quantification of the GLUT2-related transcription factor HNF1α [22,23] and analyzed the activation states of members of the insulin-independent (AMPK) and insulin-dependent (IRS-1, AKT, PKCζ) signaling pathways involved in the translocation of GLUT-4 from intracellular storage compartments to the plasmalemma [17]. In parallel, preparations obtained after cell exposure to the sole 10^−7^ M insulin were tested.

**Figure 5:**
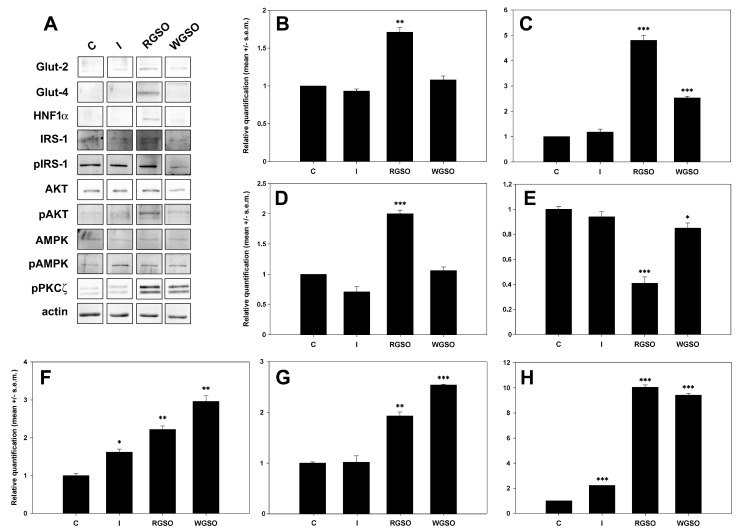
Expression of glucose transporters and their associated regulatory molecules in HepG2 cells treated with 10^−7^ M insulin (I), RGSO, or WGSO MNIDs for 24 h. Panel of immunodetected protein bands (**A**) and quantitative analysis of GLUT-2 (**B**), GLUT-4 (**C**), HNF1α (**D**), Ser307-pIRS1/IRS1 ratio (**E**), pAKT/AKT ratio (**F**), pAMPK/AMPK ratio (**G**), and pPKCζ (**H**). Each blot underwent actin loading control assessment, with one example displayed in the panel. The error bars correspond to the standard error of the mean (s.e.m.) of three independent measurements. * *p* < 0.05, ** *p* < 0.01, and *** *p* < 0.001 compared to the untreated control (C). Normality test passed.

Figure 5 displays a representative panel of the immunodetected proteins and the quantification results assessed through densitometric analysis performed with ImageJ software (Version 1.52a). The whole original images of the electrophoretic gels and blots are presented in Figure S2. The results show that WGSO specifically promoted the up-regulation of GLUT-2 (+1.5 ± 0.1-fold to the control), while RGSO enhanced the expression of both GLUT-2 (+0.7 ± 0.1-fold) and, to a greater extent, GLUT-4 (+3.8 ± 0.3-fold to the control). Furthermore, RGSO, unlike WGSO, also increased the expression of the HNF1α transcription factor (+1 ± 0.1-fold to the control), which was expected. Conversely, insulin-treated cells showed no significant changes.

Dealing with GLUT-4 stimulatory signalization, the findings demonstrate that both insulin-dependent and -independent pathways were activated by exposure to oils. In fact, the Ser307-pIRS1/IRS1 ratio decreased by about 15% and, more consistently, by about 59% to controls after exposure to WGSO and RSGO, respectively, thereby suggesting the activation of the PI3K signal transduction mechanism. This was confirmed by the up-regulation of both the pAKT/AKT ratio (+2.2 ± 0.1-fold for RGSO and +3.0 ± 0.1-fold for WGSO) and pPKCζ levels (+9.0 ± 0.2-fold for RGSO and +8.4 ± 0.1-fold for WGSO) compared to controls. Additionally, AMPK activation was also proven to contribute to the oil-induced promotion of glucose uptake, as evidenced by the up-regulation of the pAMPK/AMPK ratio by +0.9 ± 0.1-fold for RGSO and +1.5-fold for WGSO vs. the control. Compared to oil-treated cells, insulin-treated cells demonstrated a consistent, albeit lower, rise in the pAKT/AKT ratio (+0.6±0.1 fold to the control) and pPKCζ level (+1.2-fold vs. the control), although the Ser307-pIRS1/IRS1 ratio displayed only a non-significant decreasing trend.

## 3. Discussion

Various applications have been proposed for the effective management of grape seed oil waste, such as the production of biofuels and biodiesel, the manufacture of thermal insulation material, and the extraction of bioactive compounds for nutritional and biomedical uses [9,24,25,26]. In this latter context, the development of targeted therapies demands the execution of pre-clinical studies designed to elucidate the molecular and cellular scenario influencing the activity of the preparations. This investigation sought to determine if the by-products of a Sicilian wine industry, i.e., RGSO and WGSO, could positively influence glucose metabolism in cultured liver cells. The findings obtained show that both oils have substantial potential as anti-diabetic solutions, despite employing somewhat distinct molecular strategies.

In fact, even in the absence of insulin, RGSO and WGSO acted as effective inducers of glucose absorption and glycogen synthesis in HepG2 cells to a comparable degree. On the other hand, analysis of components of the cellular signaling pathways in response to treatments revealed that RSGO elevated both GLUT-2 and GLUT-4 levels, whereas the WGSO-induced rise in glucose uptake could be ascribed solely to the up-regulation of GLUT-4. GLUT-2 up-regulation in RGSO-exposed cells was coupled with a higher presence of the HNF1α transcription factor, recognized as one of the key players in the regulation of *GLUT2* expression both in vitro and in vivo [11,27]. Exposure to the oils’ MNIDs appeared to drive an increase in the synthesis of GLUT-4 and to stimulate the signaling pathways that control its translocation.

In particular, both the insulin-mediated PI3K pathway and insulin-independent AMPK pathway were activated. In addition, the participation of pPKCζ, a recognized inducer of GLUT-4 phosphorylation and actin cytoskeleton remodeling [13,14], in the oil-stimulated GLUT-4 exposure and glucose uptake was also conceivable. The administration of insulin did not result in any significant changes in the expression levels of GLUT-2, HNF1α, and GLUT-4, as well as, expectedly, in the pAMPK/AMPK ratio. In addition, a non-significant downward trend in the pIRS/IRS1 ratio and a weak increase in the pAKT/AKT ratio and pPKCζ level were found. Such limited activation of the intracellular signaling pathway, along with the observed lower rate of glucose uptake, may account for the insulin resistance that is documented to occur after a 24 h exposure of HepG2 cells to the hormone at the concentration used in this study [28].

The extraction method employed significantly influences the levels of major bioactive compounds obtained from grape seeds, and it is also well established that each grape variety, along with the oil produced, has its own unique and specific phytochemical characteristics [4,5,7]. The amount and nature of fatty acids detected in the two oil extracts here examined are in general consistent with the data reported in the literature [5,29], while their polyphenol profiles were different from those reported previously [6]. Moreover, the levels of carotenoids and chlorophyll present in RGSO and WGSO were significantly less than those reported by Laqui-Estaña et al. [30] and Fernandes et al. [31] for other types of grape seed oil extract. Of note, a low concentration of chlorophyll is an indicator of the quality of the preparations, as it is known to act as a pro-oxidant that negatively impacts oil stability and shelf-life [32]. Although the detailed mechanisms through which the oils exert their effects remain to be fully elucidated, the literature, as outlined in Table 4, suggests that certain polyphenolic components may be associated with the glucose-lowering effect on HepG2 and, more generally, liver cells.

In conclusion, our study points to RGSO and WGSO as natural agents that can influence glucose uptake by acting on GLUT transporters and components of the associated pathways. The bioactive substances present in these preparations reveal encouraging therapeutic prospects for the prevention and treatment of glucose metabolism-related disorders. Future studies will employ animal models and clinical data to thoroughly evaluate the oil’s safety profile and efficacy in managing elevated glucose levels.

The by-products of winemaking hold substantial promise for application as ingredients in the biomedical and food industry [43,44]. Given the pressing requirement for alternative treatment options against diabetes mellitus, our findings support the idea that RGSO and WGSO, or their derivatives, are valuable candidates for the development of novel agents for hyperglycemia management and beneficial supplements for the formulation of functional foods.

## 4. Materials and Methods

### 4.1. Oil Extraction and Phytochemical Analyses

By-products from red grapes of the Sangiovese variety and a white grape blend, which includes 26% Cataratto, 54% Insolia, and 20% Grillo, were provided by a Sicilian wine company. The grapes used in this study originated from western Sicily, Italy. All grape varieties were grown using organic practices in the provinces of Trapani, Agrigento, and Palermo, at altitudes ranging from 100 to 250 m above sea level. The vineyards were exposed to sunny conditions, mild temperatures, and moderate ventilation, providing optimal growth conditions. Grapes were harvested in September 2020, at technological ripeness, and processed for wine production.

#### 4.1.1. Fatty Acids, Carotenoids, and Chlorophyll

The extraction of oil from grape seeds was carried out in a Soxhlet apparatus using 300 mL of hexane as the solvent and was conducted at 60 °C for 8 h. Prior to the extraction process, grape seeds were dried in an oven at 60 °C and crushed by a grinder (multifunctional grinder; BioloMix, Fort Myers, FL, USA). The extracted oil samples were subjected to analysis for fatty acid composition, carotenoid, chlorophyll, and polyphenol content as described by Baydar et al. [45], Kapcsándi et al. [46], and Marino et al. [47]. The fatty acid profiles of the samples were investigated by gas chromatographic analysis of their methyl esters obtained through the trans-esterification process. Briefly, in a 10 mL screw-top test tube, 2 mL of hexane was added to 0.1 mL of oil sample. The mixture was shaken with a vortex for 30 s. After, 0.2 mL of 2 M methanolic potassium hydroxide solution was added, and the mixture was shaken for 30 s. The mixture was left to stratify until the upper solution became clear. The upper layer containing the methyl esters was carefully sampled by syringe and injected into the gas-chromatograph (FOCUS™ gas chromatograph; Thermo Fisher, Milan, Italy) equipped with a flame ionization detector and a fused-silica capillary column Mega-10 (50 m × 0.32 mm I.D.; film thickness 0.25 μm). The GC temperature progression was as follows: initial isotherm at 115 °C for 5 min, increase at a rate of 1.5 °C per minute from 115 to 230 °C, and final isotherm at 230 °C for 2 min. The identification of FAME peaks utilized retention time comparisons with established standards (Supelco 37 Component FAME mix; 47885-U, Supelco, Bellefonte, PA, USA). The relative abundance of FAMEs was calculated as percentage of total fatty acids, employing nonadecanoic acid (C19:0) as the internal standard for reference.

For the quantification of carotenoids and chlorophyll, RGSO and WSGO were dissolved in hexane and subjected to spectrophotometric evaluation at λ = 476 nm (for carotenoids) or 670 nm (for chlorophyll).

#### 4.1.2. Phenolic Compounds

The extraction of polyphenols from RGSO and WGSO was performed on 1 mL of each oil using 1 mL of a hydroalcoholic solution (MeOH/H_2_O, 60/40, *v*/*v*) for three consecutive cycles. The samples were then dried employing a Genevac EZ plus (StepBio, Bologna, Italy), and once the solvent evaporated, they were reconstituted in 500 μL of ACN/H_2_O (50/50, *v*/*v*) to prevent the artificial formation of secoiridoid isomers due to the presence of MeOH [48].

To identify polyphenols in the oil extracts, a UHPLC–MS/MS analysis was performed using a Vanquish Horizon instrument coupled to a Quantis Plus triple quadrupole mass spectrometer (Thermo Fisher, San José, CA, USA). The chromatographic separation was conducted using a Hypersil GOLD C18 reversed-phase column (2.1 × 50 mm, 1.9 μm particle size, Thermo Fisher), which was maintained at 30 °C and had an injection volume of 5 μL. The analysis was performed using a gradient system that combined two mobile phases: phase A, consisting of ultrapure water with 0.1% formic acid (LC–MS grade, Sigma-Aldrich, St. Louis, MO, USA), and phase B, composed of methanol (LC–MS grade, Sigma-Aldrich), with a constant flow rate of 300 μL/min. The gradient profile was structured as follows: from 0 to 2 min, 10% B; from 2 to 10 min, a linear increase to 100% B; from 10 to 12 min, followed by an isocratic hold at 100% B from 10 to 17 min. At 17.0–17.1 min, the percentage of B was linearly reduced to 10%, with a final hold at 10% B from 17.1 to 22 min. The mass spectrometer (QqQ) (Thermo Scientific) featured a heated electrospray ionization (HESI) source, and data acquisition was performed in negative ion mode. LC–MS/MS analyses were also carried out in negative ion mode, with instrument tuning performed using standard solutions of each analyte at a concentration of 1 ppm in methanol. The mass spectrometry analyses were performed under the following experimental conditions: HESI (-), spray voltage (static) 2500 V, auxiliary gas pressure 10 a.u., sheath gas pressure: 50 psi, sweep gas: 1 a.u, ion transfer tube 325 °C, vaporizer temperature 350 °C, dwell time: 100 ms, Q1 resolution:1, Q3 resolution:0.4, and CID gas (Ar) 2.0 mTorr. Selected Reaction Monitoring (SRM) was carried out on the deprotonated molecules of each polyphenol ([M-H]⁻), with the specific SRM transitions outlined in Table S1. The quantification approach relied on the integration of peak areas for all monitored transitions. For quantification, the following pure standards were used: Apigenin 7-Glucoside, Apigenin, Quercetin, Gallic Acid, L-Mandelic Acid, Chlorogenic Acid, hydroxycinnamic acid, kaempferol, Caffeic Acid, Catechin, Epicatechin, Rutin, Cumaric Acid, syringic acid, Gentisic Acid, Ferulic Acid, Luteolin, olacein, Oleocanthal, and hydroxytyrosol. An external calibration method was employed to quantify phenolic compounds. A stock solution in methanol was prepared at a concentration of 5 µg/mL for each analytical standard. From this, five additional calibration solutions were prepared at concentrations of 1 µg/mL, 500 ng/mL, 100 ng/mL, 50 ng/mL, and 5 ng/mL per analyte. The calibration curve showed a linear correlation coefficient (R^2^) of 0.99. Data processing was performed using the Quan/Qual Browser Trace Finder software (Version 3.3, Thermo Fisher Scientific). Each calibration point was determined as the average of three independent injections. The limits of detection (LODs) and quantification (LOQs) for each compound in the standard solutions were estimated based on the blank signal and the regression curve, averaging five blank injections interposed with standards. The LOD was defined as the concentration producing a signal equivalent to the blank signal plus three times its standard deviation, while the LOQ was calculated as the blank signal plus ten times its standard deviation, ensuring reliable quantification.

### 4.2. Cell Culture and Treatments

HepG2 liver tumor cells, derived from the liver tissue of a 15-year-old white male with a hepatoblastoma [49], were thawed from frozen laboratory stocks and routinely maintained in high glucose–DMEM medium (D6429, Sigma-Aldrich) supplemented with 10% heat-inactivated fetal bovine serum (FBS; F4135, Sigma-Aldrich) and antibiotics (100 U/mL penicillin and 100 g/mL streptomycin; Capricorn Scientific GmbH, Ebsdorfergrund, Germany) in a humidified incubator at 37 °C and a 5% CO_2_ atmosphere. Stock solutions of either WGSO or RGSO were prepared by dissolving the oil in FBS; cell treatments were performed with a range of dilutions of the stock solutions in complete DMEM medium.

### 4.3. Viability Assay

HepG2 cells were seeded at a density of 7000 cells/well in 96-well plates and were treated with different dilutions of WGSO and RGSO. Untreated cells were used as controls. Following the addition of MTT and subsequent cell lysis, the optical density of the dissolved formazan was measured at a λ = 550 nm [50]. Cell viability was quantified as a percentage relative to control, allowing for the assessment of the minimum non-inhibitory dilution (MNID) of the oils, i.e., the lowest final dilution that did not compromise cell viability, if any, which was then used in subsequent biological assays.

### 4.4. PAS Staining

HepG2 cells were plated at a density of 88,000 cells/well in 6-well plates. When the cells became subconfluent, they were treated with the MNIDs of either WGSO or RGSO, with or without the addition of 10^−7^ M insulin (Santa Cruz Biotechnology, Heidelberg, Germany), or solely with 10^−7^ M insulin for 24 h. Untreated cells were used as controls. The staining of intracellular glycogen was achieved through the PAS reaction involving fixation with 4% paraformaldehyde solution (Santa Cruz Biotechnology) and treatment with 0.5% periodic acid solution (Santa Cruz Biotechnology) followed by exposure to Schiff’s reagent (Santa Cruz Biotechnology) [51]. Glycogen deposits were visualized under the light microscope as pink precipitates and photographed. The average staining density, in relation to the area covered by cells, was computed using ImageJ software with three independent measurements taken for each preparation, and the results were plotted in a bar graph.

### 4.5. Evaluation of Glucose Consumption and Uptake

As previously reported [51], for the evaluation of glucose consumption, HepG2 cells were seeded at a concentration of 40,000 cells/well in 24-well plates. When the cells became subconfluent, they were treated with the MNIDs of either WGSO or RGSO, with or without the addition of 10^−7^ M insulin (Santa Cruz Biotechnology), or solely with 10^−7^ M insulin for 24 h. Untreated cells were assayed as controls. After incubation, the glucose concentration within the diluted media was evaluated using a blood glucometer (Glucomen Areo 2k, Menarini Diagnostics, Firenze, Italy) and disposable test strips (Glucomen Areo Sensor, Menarini Diagnostics) in triplicate experiments.

For glucose uptake assessment, HepG2 cells were seeded at a concentration of 88,000 cells/well in 6-well plates. When the cells became subconfluent, they were treated with the MNIDs of either WGSO or RGSO or with 10^−7^ M insulin for 24 h. Untreated cells were assayed as controls. Upon the removal of the medium, the cells were incubated with the non-metabolizable fluorescent analog of D-glucose 2-[N-(7-nitrobenz-2-oxa-1,3-diazol-4-yl)amino]-2-deoxy-D-glucose (2-NBDG; Peptide Institute, Osaka, Japan) for 1h before undergoing flow cytometric analysis to assess its uptake in the fluorescein isothiocyanate (FITC) channel. This evaluation utilized a FACSCanto flow cytometer (BD Biosciences, Franklin Lakes, NJ, USA) and involved the analysis of 10,000 individual cell events. Unstained controls were analyzed in parallel for the control of the background derived from autofluorescence. The analysis of the .fcs files obtained involved measuring the mean FITC intensity of the positive fractions through the Floreada tool, which is available online at https://floreada.io (accessed on 2 October 2024), and the average data were plotted in a bar graph. Concurrently, a propidium iodide (PI) stain was performed to evaluate whether the treatment-induced any cell death.

### 4.6. Western Blot

As previously reported [52], control and treated HepG2 cells underwent harvesting and lysis in a buffer solution that contained 7 M Urea, 2% CHAPS, 10 mM DTT, and a protease inhibitor cocktail. Protein samples were subjected to electrophoresis on 13% SDS-PAGE and subsequently transferred onto nitrocellulose membranes. The membranes were probed overnight at 4 °C with different rabbit primary antibodies, including anti-GLUT2 (bs-10379R-TR, Bioss, Boston, MA, USA; working dilution 1:500), anti-GLUT4 (bs-0384R-TR, Bioss; working dilution 1:500), anti-HNF1α (PAG775Hu01, Cloud-Clone Corp., Katy, TX, USA; working dilution 1:1000), anti-AKT (9272, Cell Signaling Technology, Danvers, MA, USA; working dilution 1:750), anti-pAKT (sc-7985-R, Santa Cruz Biotechnology, working dilution 1:500), anti-IRS1 (NB-22-3767, NeoBiotech, Nanterre, F, working dilution 1:1000), anti-pIRS1 Ser307 (NB-22-0306, NeoBiotech, working dilution 1:1000), anti-AMPK (CPA9232, Cohesion Biosciences, working dilution 1:1000), anti-pAMPK Thr183/172 (NB-22-0735-50, NeoBiotech, working dilution 1:1000), anti-pPKCζ Thr410 (MA5-46896, Invitrogen, Waltham, MA, USA, working dilution 1:1000) and, as an internal control, anti-actin (NB-22-1460, NeoBiotech; working dilution 1:1000). After a 1 h incubation at room temperature with the peroxidase-conjugated secondary antibody (Ab6721, Abcam; working dilution 1:3000), an enhanced chemiluminescence system (Versadoc MP Imaging System, Bio-Rad, Hercules, CA, USA) was employed to visualize the immunoreactions, utilizing the SuperSignal West Pico Plus substrate (Thermo Fisher). The quantification of signal intensity involved the use of Quantity One (Bio-Rad, Hercules, CA, USA) or ImageJ software, with normalization based on the intensity of the actin band.

### 4.7. Statistics

One-way ANOVA and normality tests were performed using SigmaPlot 11.0 software (SYSTAT, San Jose, CA, USA). For the Western blot experiments, the analysis involved a two-tailed unpaired Student’s *t*-test performed with GraphPad Prism 9 software (GraphPad, San Diego, CA, USA).

## Figures and Tables

**Table 1:** Fatty acid composition (mean %) of WGSO and RGSO samples.

Fatty Acid	WGSO	RGSO
C14:0	0.1	0.1
C16:0	8.0	7.8
C16:1 ω7 cis	0.04	0.03
C17:0	0.1	0.05
C18:0	4.1	4.2
C18:1 ω9 cis	19.9	16.8
C18:1 ω7 cis	1.2	1.2
C18:2 ω6,9 cis, cis	65.7	68.8
C20:0	0.1	0.1
C18:3 ω3 + C20:1 ω9	0.5	0.5
C20:2	nd	0.03
C22:0	nd	0.03

nd = not determined.

**Table 2:** Carotenoid and chlorophyll content (mg/Kg) in WGSO and RGSO samples.

	WGSO	RGSO
Carotenoids	0.02	0.2
Chlorophyll	nd	0.02

nd = not determined.

**Table 3:** Polyphenol compounds detected in WGSO and RGSO samples (ng/g).

Polyphenol	WGSO	RGSO
Hydroxytyrosol	<LOQ	<LOQ
Cumaric Acid	<LOQ	39.5
Ferulic Acid	24.0	28.0
Olacein	10.5	<LOQ
Oleocanthal	20.1	<LOD
Syringic Acid	153.2	4213.3
Trans-OH-Cinnamic	61.4	323.5
Rutin	<LOQ	<LOQ
Kaempferol	163.1	120.9

LOQ: limit of quantification; LOD: limit of detection.

**Table 4:** Biological properties of polyphenols present in RGSO and WGSO.

Molecule	Bioactivity	References
Cumaric acid	Increase of GLUT-2 expression	[33]
	Activation of PI3K-AKT pathway in vivo	[34]
Ferulic acid	AMPK activation and increase of GLUT-2 and GLUT-4 expression	[35,36]
	Activation of PI3K-AKTpathway	[37]
Kaempferol	AKT activation in vitro and in vivo	[38,39]
Rutin	AKT activation in vivo	[40]
Syringic acid	Increase of glucose utilization by peripheral tissues	[41,42]

## Data Availability

All data generated or analyzed during this study are included in this published article.

## Supplementary Materials

The following supporting information can be downloaded at: https://www.mdpi.com/article/10.3390/molecules30091933/s1, Figure S1: Representative PAS staining images showing glycogen accumulation in HepG2 cells grown in the different experimental conditions; Figure S2: Images depicting the complete protein blots; Table S1: UHPLC-MS/MS parameters and limit of quantification for phenolic compounds identified in grape seed oil extracts.
