# Supplementary material for: Valorization of Winemaking By-Products: White and Red Grape Seed Oils Improve Glucose Consumption and Uptake In Vitro
Source: Molecules. 2025 Apr 26;30(9):1933. doi: 10.3390/molecules30091933 (PMC12073708; doi:10.3390/molecules30091933)
Supplement: Supplementary file 1 [file molecules-30-01933-s001.zip › molecules-3577156-supplementary.pdf]

Valorization of winemaking by-products: white and red grape seed oils improve glucose consumption and uptake *in vitro*

Daniela Ganci, Federica Bellistrì, Manuela Mauro, Roberto Chiarelli, Francesco Longo, Serena Indelicato, Sergio Indelicato, Vito Armando Laudicina, Vincenzo Arizza, Mirella Vazzana and Claudio Luparello

SUPPLEMENTARY MATERIAL

Figure S1: Representative PAS staining images showing glycogen accumulation in HepG2 cells grown in the different experimental conditions. Stained preparations of control and treated cells were examined under the light microscope, photographed, and processed for quantification of the mean density values using ImageJ software, as presented in Fig.2. Microscopic magnification = 20x.

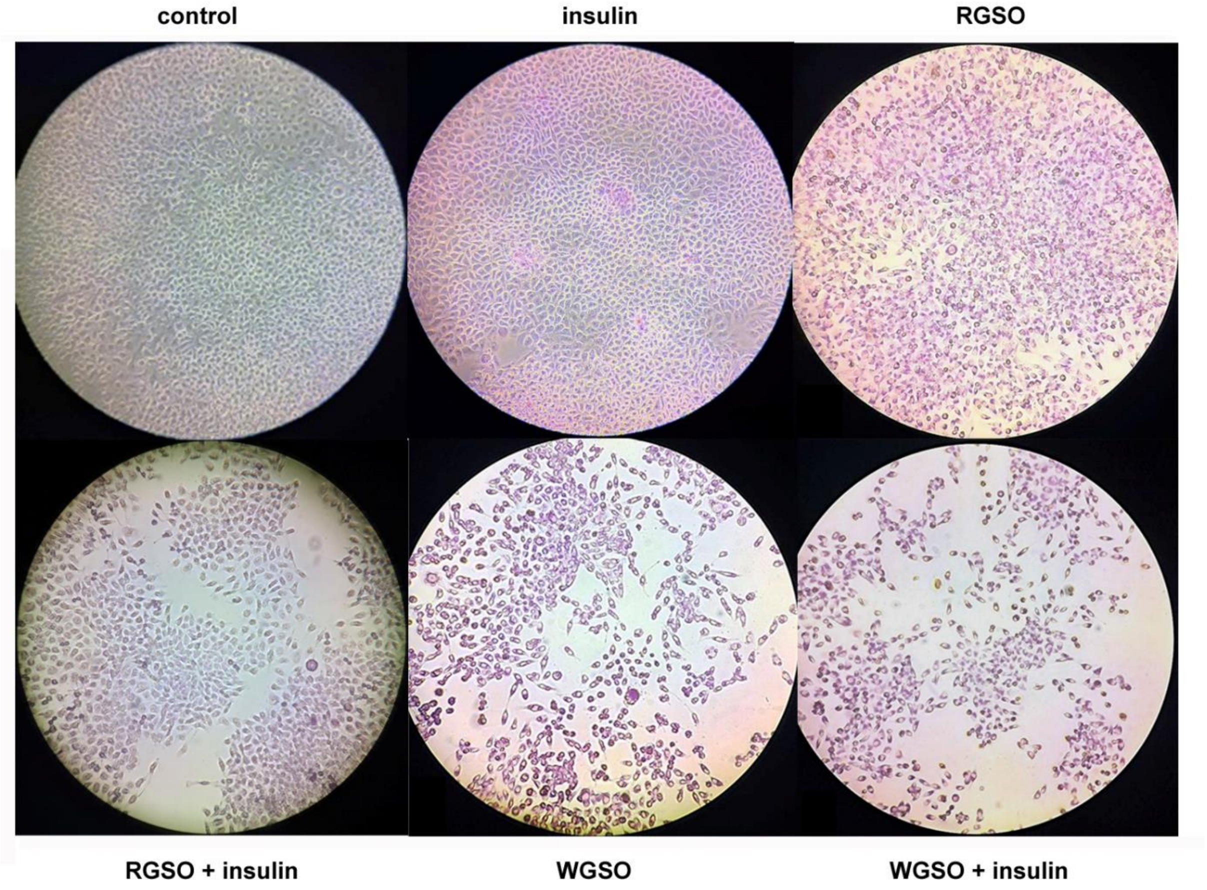

Figure S2: Left: Images depicting the complete protein blots stained with Ponceau S, as well as the molecular weight markers for reference. Right: Images depicting the corresponding immunoblots from which the edited inserts have been utilized for the figures presented in the paper. Control = **sample 1**, RGSO = **sample 2**, WGSO = **sample 3**, Control + Insulin = **sample 4**, RGSO + Insulin = **sample 5**, WGSO + Insulin **= sample 6**.

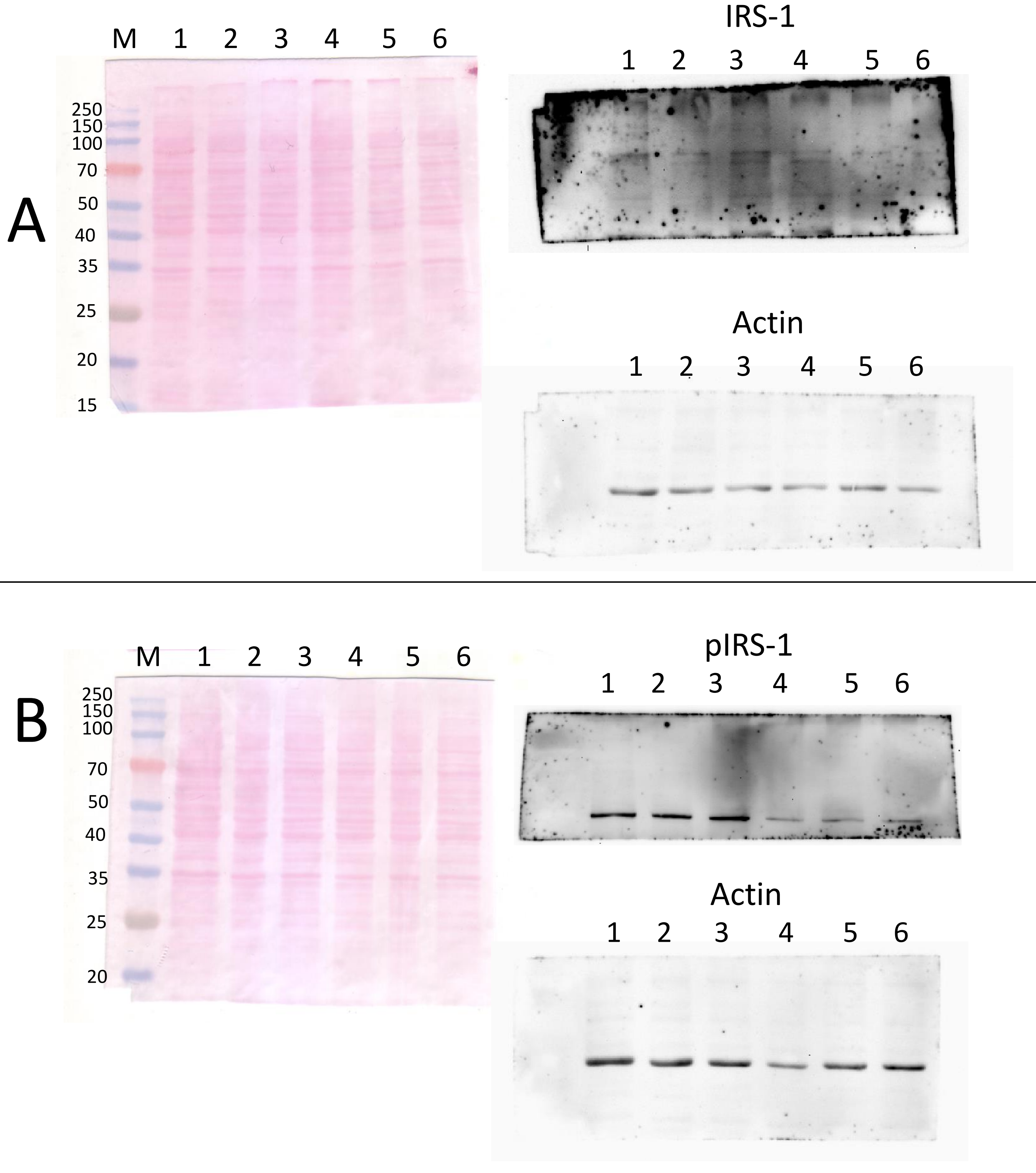

C

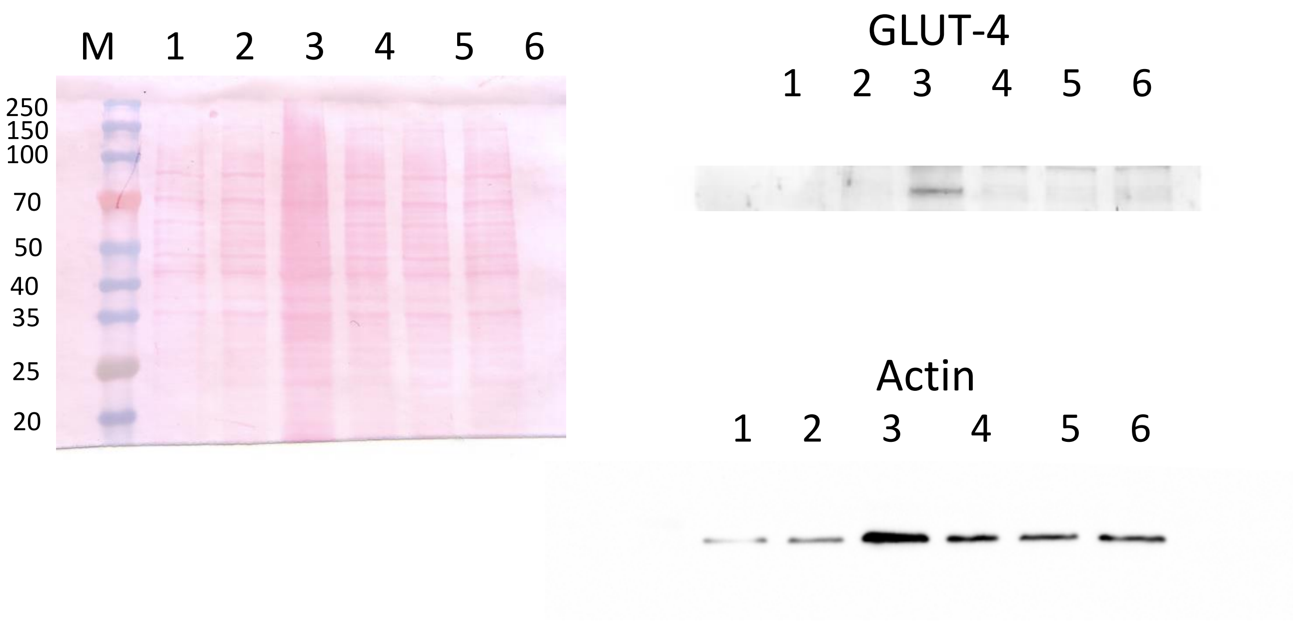
M 1 2 3 4 5 6
250
150
100
70
50
40
35
25
20
GLUT-4
1 2 3 4 5 6
Actin
1 2 3 4 5 6

D

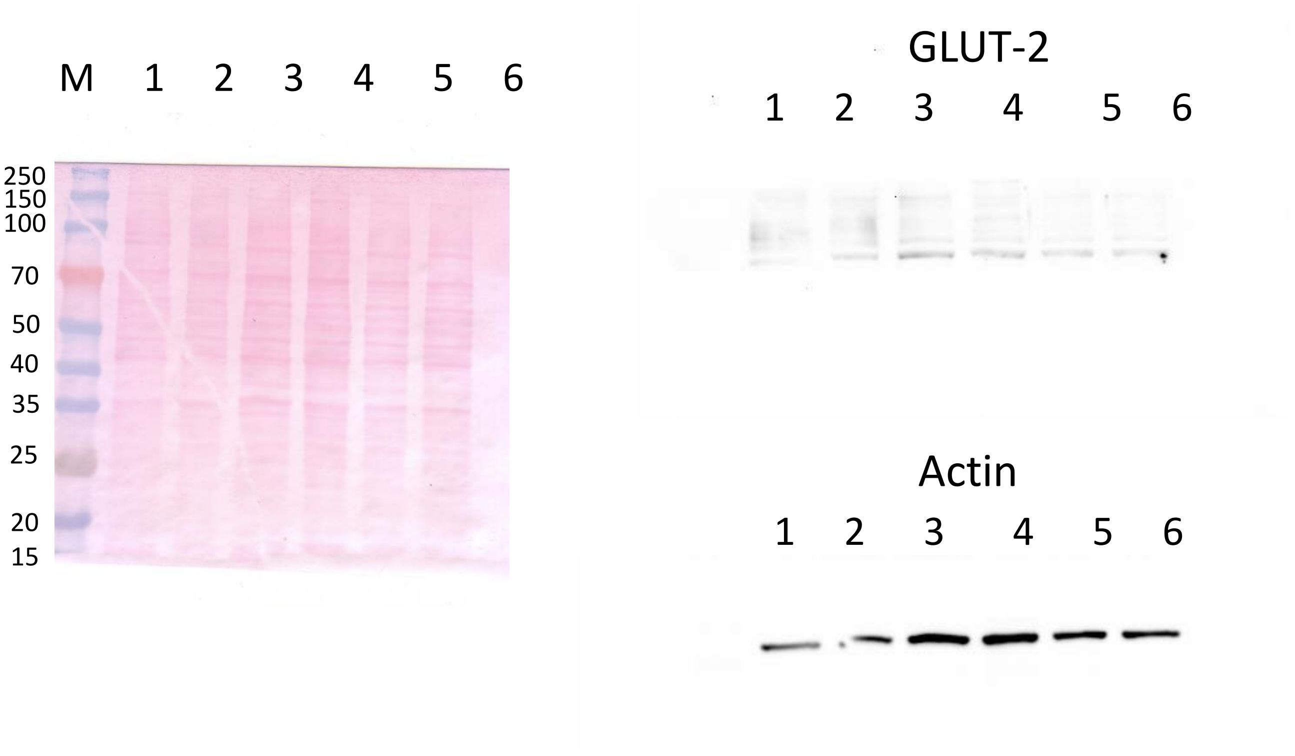
M 1 2 3 4 5 6
250
150
100
70
50
40
35
25
20
15
GLUT-2
1 2 3 4 5 6
Actin
1 2 3 4 5 6

E

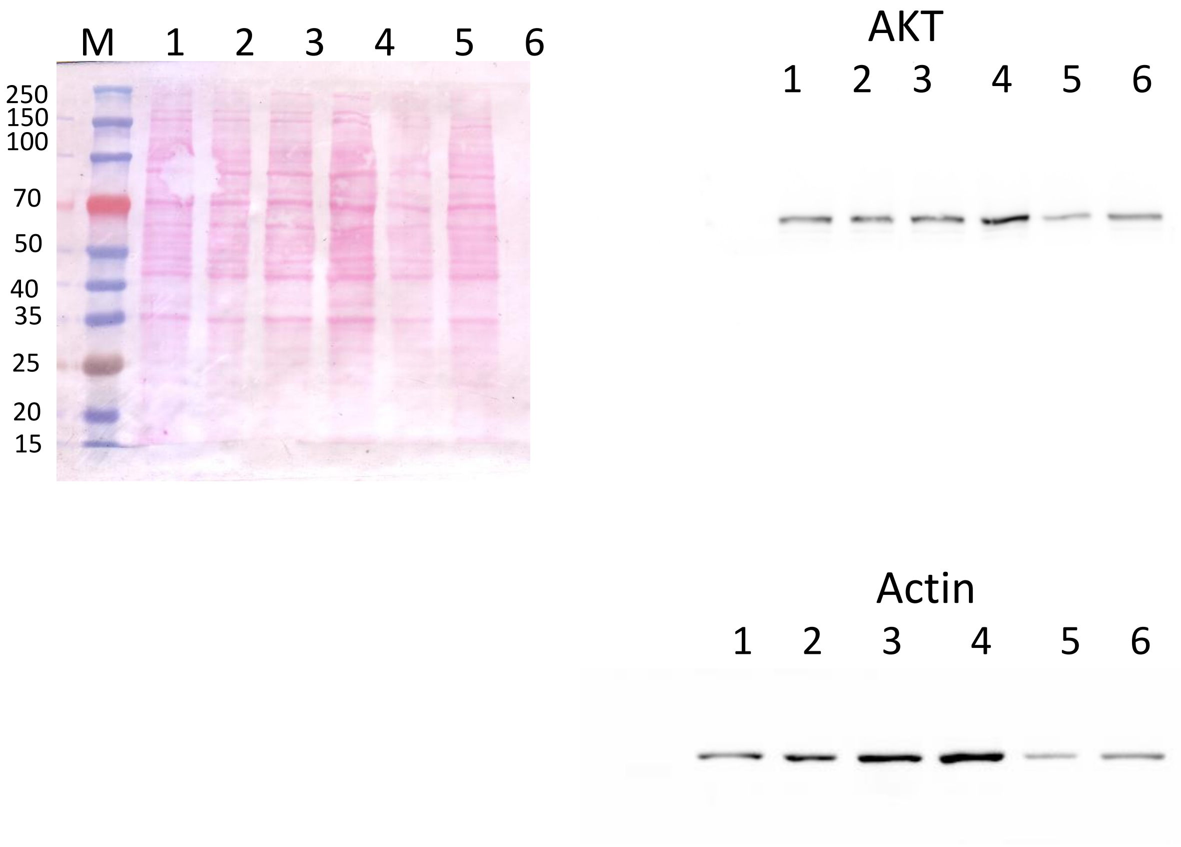
M 1 2 3 4 5 6
250
150
100
70
50
40
35
25
20
15
AKT
1 2 3 4 5 6
Actin
1 2 3 4 5 6

F

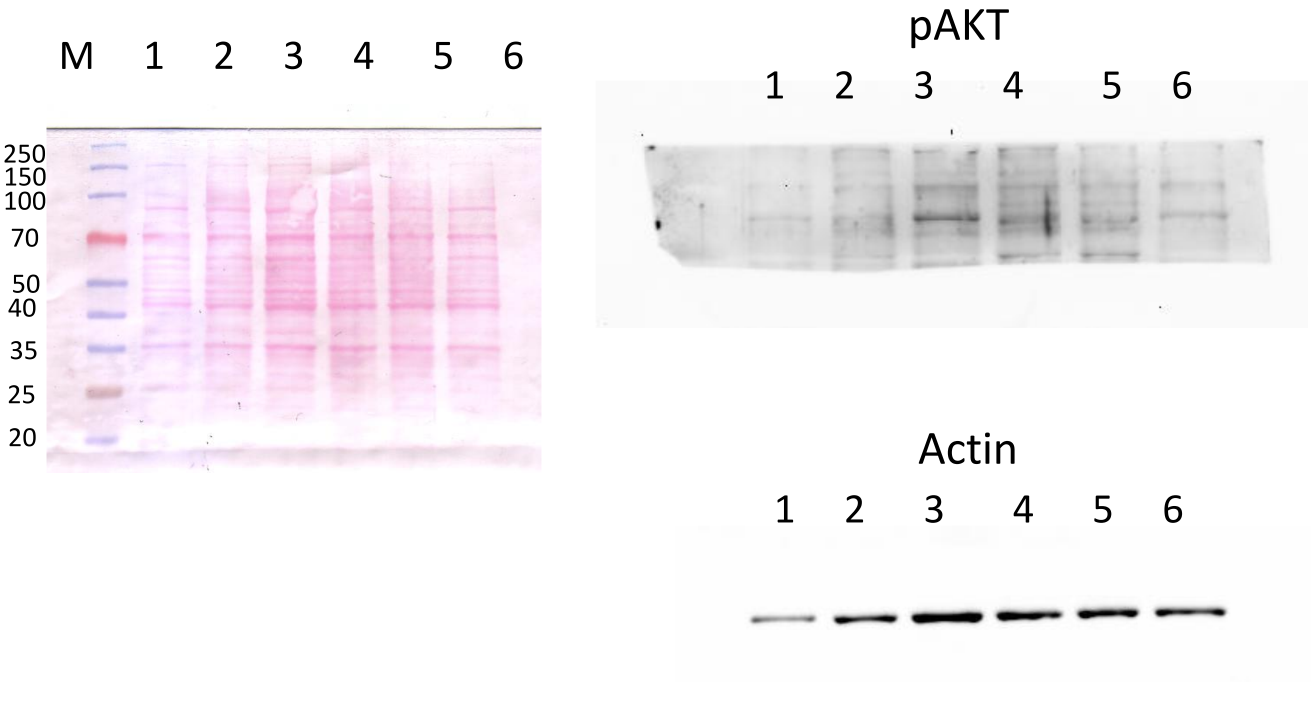
M 1 2 3 4 5 6
250
150
100
70
50
40
35
25
20
pAKT
1 2 3 4 5 6
Actin
1 2 3 4 5 6

G

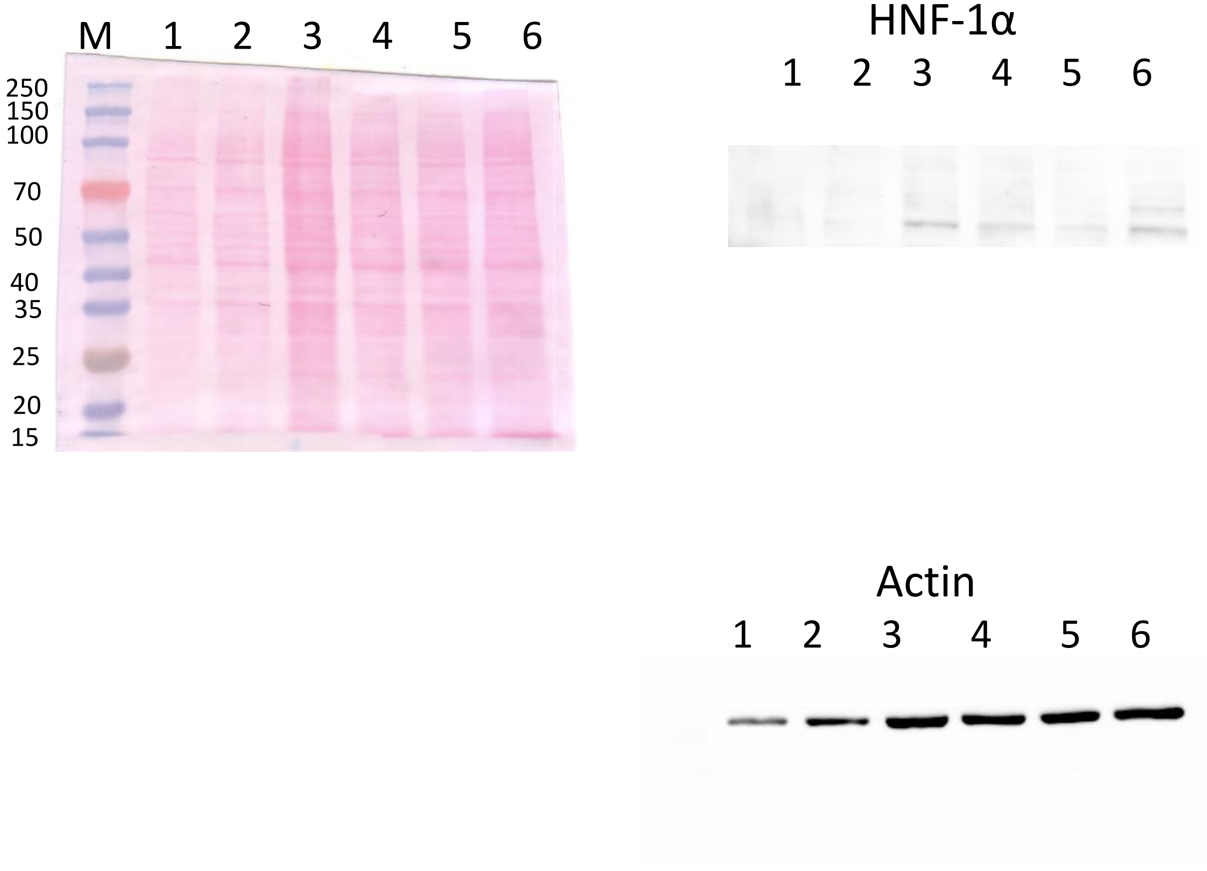
M 1 2 3 4 5 6
250
150
100
70
50
40
35
25
20
15
HNF-1α
1 2 3 4 5 6
Actin
1 2 3 4 5 6

H

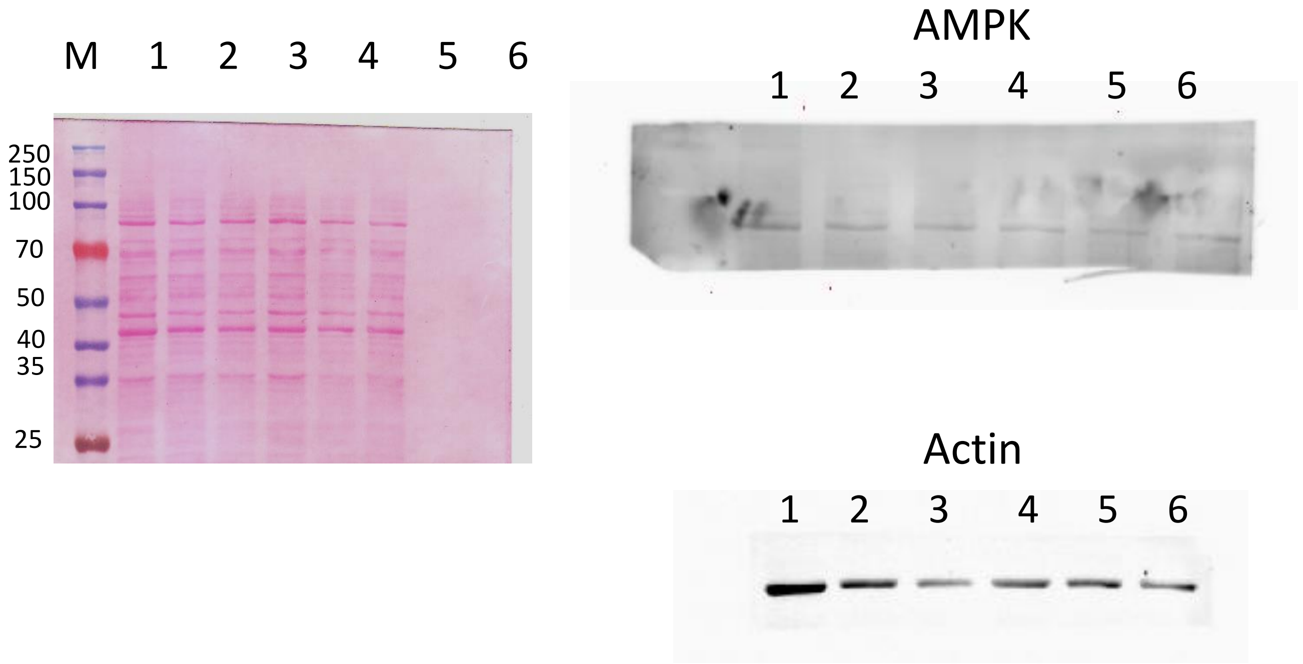
M 1 2 3 4 5 6
250
150
100
70
50
40
35
25
AMPK
1 2 3 4 5 6
Actin
1 2 3 4 5 6

I

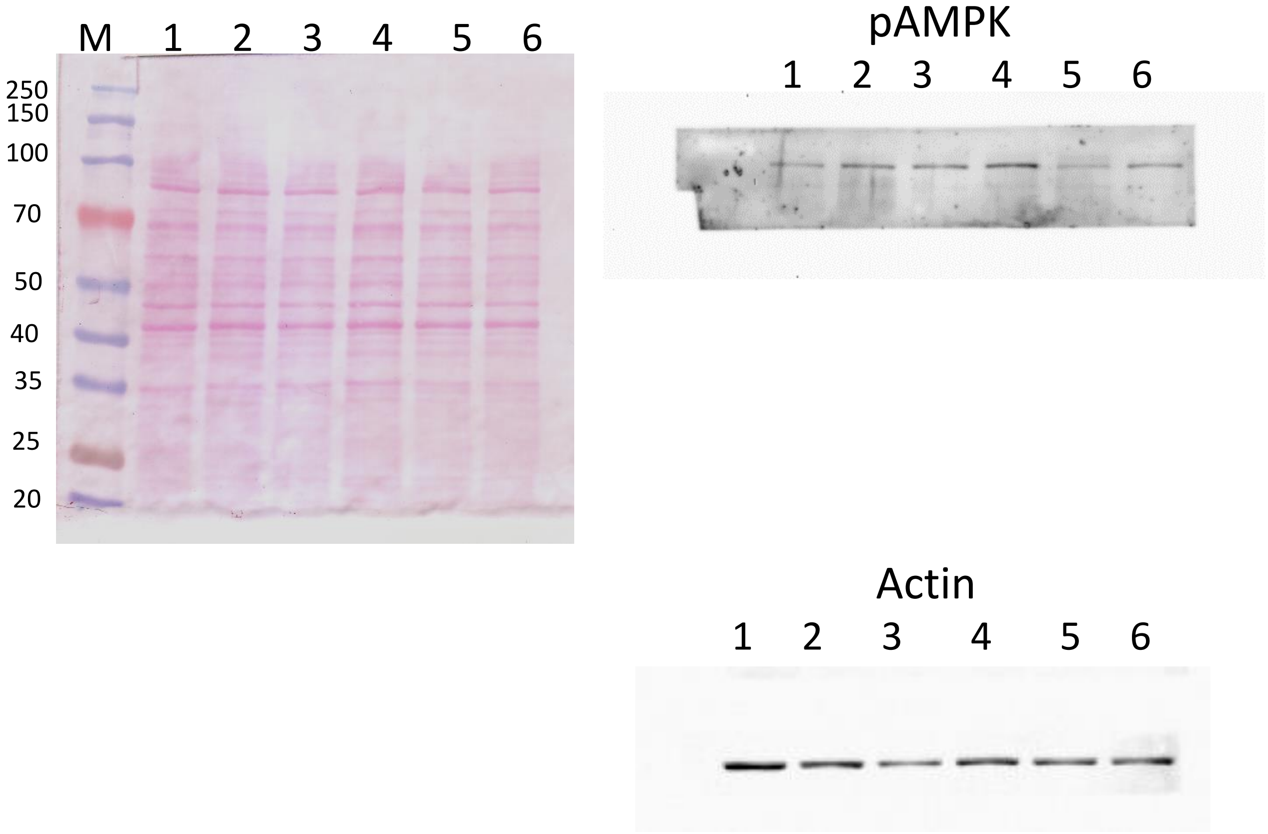
M 1 2 3 4 5 6
250
150
100
70
50
40
35
25
20
pAMPK
1 2 3 4 5 6
Actin
1 2 3 4 5 6

L

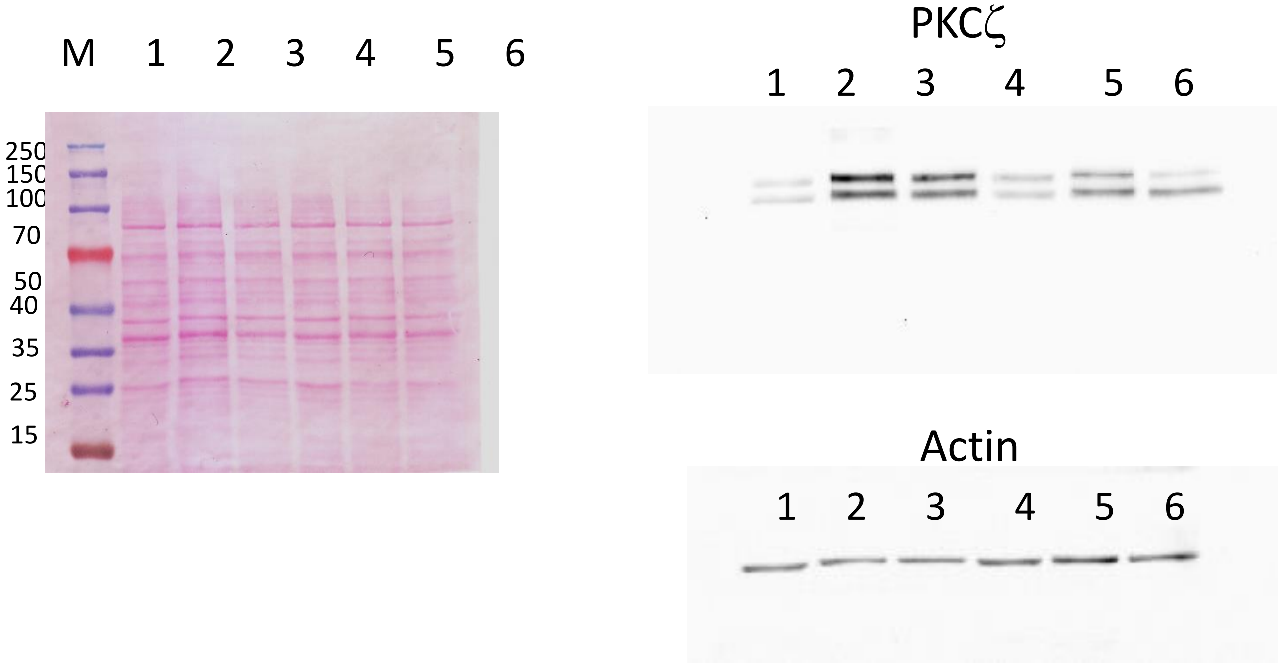
M 1 2 3 4 5 6
250
150
100
70
50
40
35
25
15
PKCζ
1 2 3 4 5 6
Actin
1 2 3 4 5 6

Table S1: UHPLC-MS/MS parameters and limit of quantification for phenolic compounds identified in grape seed oil extracts

	Precursor Ion (m/z) [M-H]⁻	Product Ion (m/z)	Collision Energy (V)	RF Lens (V)	LOQ (µg/L)
Gallic Acid	169	79	24	101	30
	169	125	14	101	
Ferulic Acid	193	134	15	99	18
	193	178	13	99	
Chlorogenic Acid	353	179	45	180	26
	353	191	45	180	
Catechin	289	203	20	147	28
	289	245	15	147	
Mandelic Acid	151	77	18	65	20
	151	107	10	65	
Gentisic Acid	153	108	22	90	20
	153	109	14	90	
Syringic Acid	197	153	12	100	22
	197	182	14	100	
Caffeic Acid	179	107	25	101	20
	179	135	16	103	
Trans-OH-Cynnamic	163	93	31	90	27
	163	119	14	90	
Rutin	609	271	60	299	20
	609	300	38	299	
Resveratrol	227	143	27	156	20
	227	185	20	156	
Apigenin-7Glu	433	269	20	123	18
	433	271	20	123	
Quercetin	301	151	18	166	20
	301	179	21	166	
Kaempferol	285	202	20	195	20
	285	239	29	195	
Hydroxytyrosol	153	95	21	97	10
	153	123	14	97	
Cumaric Acid	163	93	31	91	27
	163	119	13	91	
Luteolin	285	133	35	187	10
	285	175	26	187	
Apigenin	269	117	35	178	10
	269	151	25	178	
Olacein	319	165	5.4	178	10
	319	195	5.4	178	
Oleocanthal	303	165	5.4	195	10
	303	285	8.4	195	
